# Established and Nascent Entrepreneurs: Comparing the Mental Health, Self-Care Behaviours and Wellbeing in Singapore

**DOI:** 10.3389/fsoc.2022.843101

**Published:** 2022-03-25

**Authors:** Jiankun Gong, Zezheng Xu, Sherry Xueli Wang, Mingyan Gu, PuayChin Ong, Yuanzhe Li

**Affiliations:** ^1^Faculty of Art and Social Science, University of Malaya, Kuala Lumpur, Malaysia; ^2^Department of Diplomacy, China Foreign Affairs University, Beijing, China; ^3^Institute of Central China Development, Wuhan University, Wuhan, China; ^4^Department of Geography, National University of Singapore, Queenstown, Singapore; ^5^School of Business and Management, University of South Wales, Cardiff, United Kingdom; ^6^Stirling Management School, Singapore Institute of Management, Singapore, Singapore; ^7^School of Materials Science and Engineering, Nanyang Technological University, Singapore, Singapore

**Keywords:** mental health, Attention Deficit Hyperactivity Disorder (ADHD), dyslexia, wellbeing, Singaporean entrepreneurs

## Abstract

Mental health problems currently affect a quarter of the world's population. Recent research in western societies has started to examine the relationship between entrepreneurship and mental health problems such as Attention Deficit Hyperactivity Disorder (ADHD) and dyslexia. However, little has been done to categorize entrepreneurs into different types and investigate how their levels of mental health and well-being correspond to these types. This study divided entrepreneurs into established and nascent categories and examined this topic in Singapore. By distributing two sets of surveys, a total of 834 responses were collected, with 346 responses from established entrepreneurs and 488 responses from nascent ones. The results showed that the nascent entrepreneurs' levels of well-being were found to be much lower than those of the established entrepreneurs. Furthermore, entrepreneurs with ADHD or dyslexia symptoms generally had a much lower level of life satisfaction, compared with those without. However, the self-care behaviours observed in this sample differed somewhat from observations made in western societies, which might be explained by the different cultures and habits in Singaporean society. The findings not only highlight the need for relevant organizations to support nascent entrepreneurs but serve to remind veteran entrepreneurs to practice more healthy self-care behaviours.

## Introduction

In recent decades, as affluence levels have significantly improved and basic survival needs are no longer a concern, greater attention has been focused on people's spiritual needs and mental health. Mental health relates to people's emotional and behavioural well-being. It could have significant impacts on their daily life, work performance and relationships with others (Nordqvist, 2017). According to the World Health Organization, good mental health is an essential factor in determining whether an individual could “realize his or her own potential, cope with the normal stresses of life and work productively”. Mental health problems or disorders are defined as behaviours or mental patterns that cause significant negative impacts on normal human functioning. In fact, one in four people worldwide are affected by mental health problems such as depression, anxiety, dyslexia and Attention Deficit Hyperactivity Disorder (ADHD). Extensive scholarly research has already examined the negative impacts of mental health problems on workplace performance. According to a World Health Organization (2000), people with mental health problems may display higher error rates and absenteeism, lower productivity and efficiency, a lack of motivation and enthusiasm and poorer relationships with others. In the context of entrepreneurship, the same phenomena have also been observed. Compared to those in regular employment, entrepreneurs are usually believed to possess more positive traits, such as higher resilience, a greater ability to cope with stress and emergencies, and higher levels of efficiency and motivation. It is therefore logical to expect that mental health problems may cause even more severe harm to entrepreneurs in terms of job performance. Traditional research on entrepreneurs and their mental health conditions has generally focused on a singular group, while the differences between veteran and nascent individuals are seldom examined. Therefore, a gap exists in this area of knowledge that warrants further attention and exploration. Moreover, as entrepreneurs make very important contributions to economic development and constitute an essential part of society as a whole, it is useful to know more about their mental health and well-being and render care and support to them if necessary.

ADHD is a prevalent neurodevelopmental psychological disorder characterized by difficulties with attention, impulsivity, and activity level (DuPaul et al., 1998). Recently, researchers have started to accentuate the relationship between mental health problems and entrepreneurial intentions, specifically debating whether there is a clear relationship between entrepreneurial tendencies and ADHD and dyslexia symptoms (Alexander-Passe, 2015; Wiklund et al., 2018). It seems people with ADHD might potentially have qualities to be well suited for entrepreneurship (Wiklund et al., 2016). Extant empirical studies revealed that the clinical condition of ADHD is positively connected to both entrepreneurial intentions and initiation of business ventures and individual-level entrepreneurial orientation (Lerner et al., 2019; Thurik et al., 2020; Moore et al., 2021). Plus, the entrepreneurship environment is often characterized by high uncertainty, risks and unpredictability. Therefore, instead of merely following rules and routines, entrepreneurs are frequently required to think out of the box and take quick and intuitive decisions and actions. This seems to be reasonably compatible with certain traits that are exhibited by people with ADHD symptoms and researchers are curious about the possibility that ADHD traits could induce entrepreneurial intentions and behaviours (Verheul et al., 2015; Hatak et al., 2020). On the other hand, the context of entrepreneurship may also mean people are more likely to display behaviours associated with ADHD or dyslexia. In line with the discussion, there is close interconnectedness between ADHD, mental health and entrepreneurs, which requires further exploration of this group. Thus, this study mainly focuses on describing entrepreneurs and their mental health, self-care behaviour and wellbeing.

While researchers have started to draw attention to this topic and successful studies have already been conducted in some developed countries such as the USA and Australia (Hatak et al., 2020; Tucker et al., 2021), this topic remains in its infancy, with many different research directions worth investigating. This project examines this topic in the context of Singapore and focuses on assessing the well-being and self-care behaviours of entrepreneurs, with special attention to those with ADHD or dyslexia symptoms.

Singapore is one of the most densely populated countries and has experienced major economic development during recent decades. Meanwhile, entrepreneurship has also grown at an exponential rate (Halunko et al., 2018). This surge of entrepreneurship can be traced to the time of Lee Kuan Yew and the economic reforms of the 1990s, when the first generation of entrepreneurs ventured into businesses, many of them achieving great success. In recent years, Singapore has recognized entrepreneurship as a key driving force of national economic development, with a special emphasis on technology and innovation. With its promising and prospering entrepreneurship environment, its distinctive history and culture compared to western societies and the increasing awareness of the importance of mental health and well-being, Singapore is an appropriate country in which to study and investigate this topic. The results can be compared to those from the USA and Australia, in which previous research has been conducted. Different results might be expected due to the highly distinctive national conditions and business cultures in Singapore. Also, extant literatures suggest that the habitual/experienced entrepreneurs (i.e. those with prior business ownership experience) and novice entrepreneurs (i.e. those with no prior business experience as a founder, inheritor or purchaser of a business) are cognitive, emotional and behavioural different (Ucbasaran et al., 2003; Baron and Ensley, 2006; Engel et al., 2020). Moreover, (Westhead et al., 2005) found that similarities and differences between novice, serial and portfolio entrepreneurs are highlighted with regard to their experience and cognition. Numerous studies revealed that cognition is always associate with mental health and wellbeing (Davis et al., 2015; Haver et al., 2019; Mensmann and Frese, 2019). Thus, this study divides entrepreneurs into two categories and try to find the nuances between them.

This paper mainly consists of three parts. In the first part, relevant literature, concepts and theoretical underpinnings are clarified and the main findings of similar studies from which inspiration was drawn are summarized. In the second part, the research method is presented and the results and findings are discussed. Lastly, conclusions and recommendations based on the results and analysis are provided.

## Literature review

### Entrepreneurship, Mental Disorder, ADHD, and Dyslexia

Entrepreneurship is the act of creating and managing a new business in order to make a profit. The term is often associated with risk-taking, innovation and creativity. Unlike most regular employment, in which people have to follow a fixed schedule and complete tasks assigned by their management, entrepreneurs enjoy a very high level of autonomy and flexibility to plan their own schedules and work. This is one of the most attractive reasons why people choose to become entrepreneurs (Estrin et al., 2019).

Mental disorders are behaviours or mental patterns that cause significant negative impacts on normal human functioning. Extensive research has already examined how mental disorders could severely affect workplace performance (Tsuchiya et al., 2012; Hakulinen et al., 2019). Some of the most commonly observed mental disorders and problems are ADHD and dyslexia. ADHD is short for Attention Deficit Hyperactivity Disorder; its symptoms may include anxiety, impulsivity, hyperactivity, chronic lateness and forgetfulness. Research has found that those with ADHD are 10–14% less likely to be employed. Those who have a job tend to have a much lower income level, estimated to be 33% lower on average compared to people who do not suffer from ADHD (Fletcher, 2014). Dyslexia is a type of learning difficulty related to language learning and cognition. It mainly affects the ability to read and spell; therefore, it is also known as reading disorder (Lyon et al., 2003).

In recent years, research and literature have started to examine the positive aspects of mental disorders. Specifically, certain negative behaviours may become useful in different contexts (Wiklund et al., 2017; Wienen et al., 2019). Many similarities have been identified between ADHD symptoms and entrepreneurial behaviours (see Appendix). Some positive traits of people with ADHD include having more energy and creativity (Wiklund et al., 2017), being more willing to take risks (Wiklund et al., 2018) and making decisions quickly and decisively in uncertain environments (Archer, 2015). These traits are especially important in the entrepreneurial setting and people with ADHD could make use of their symptoms to achieve entrepreneurial success.

A study by McGowan et al. (2008) found that students with ADHD were more entrepreneurial than other university students and were more likely to be “right brain” persons, which is typical of successful entrepreneurs. The Durham University General Entrepreneurial Tendency (GET) Test was used to measure the entrepreneurial tendencies of students with ADHD and those without ADHD. It was found that students with ADHD score much higher, on average, in each test category test than those without ADHD, indicating a much stronger inclination toward entrepreneurship. This study and its findings are of great importance to entrepreneurship education and may help ADHD students to identify and exploit their potential and strong points, instead of being regarded as problem cases who always lag behind their peers.

Apart from the phenomena observed in schools, similar patterns have also been observed in the real world of entrepreneurship. Many famous and established entrepreneurs have been identified as displaying ADHD behaviours, such as Bill Gates, Donald Trump and David Neeleman (Lerner et al., 2018). Researchers have started to examine the relationship between ADHD and entrepreneurship among real business entrepreneurs (Wiklund et al., 2017; Tucker et al., 2021). It has been found that ADHD influences entrepreneurial tendencies in multi-dimensional ways (Wiklund et al., 2019). It is positively related to entrepreneurship in terms of hyperactivity, sensation seeking and a lack of premeditation. Hyperactivity allows people with ADHD to concentrate intensely and immerse themselves in their businesses for considerable periods of time (Wiklund et al., 2016). Sensation seeking means that people with ADHD are innately attracted to risky and stimulating activities. Therefore, entrepreneurship would naturally be attractive to them as it involves a much uncertainty and risk. People with ADHD are also more suited to entrepreneurship as they tend not to avoid risky ventures and opportunities, compared to the majority of people, who are generally risk averse. A lack of premeditation is typically a highly negative behaviour in normal situations. However, it is unexpectedly useful in the entrepreneurial setting, as people have to make decisions and actions very quickly to seize fleeting opportunities despite having little actual information. People with ADHD tend to act swiftly, even before thinking carefully and they trust their gut feelings and intuition. This may be a particular advantage in the entrepreneurial context. Since most people typically spend a considerable time collecting information, thinking and analyzing, they either often miss opportunities or never attempt to seize them, as they are not sufficiently confident, due to their lack of knowledge and information.

### Entrepreneurs' Wellbeing and Self Care Behaviours

It has been proven that effective self-care methods, such as getting enough sleep, exercise, yoga and meditation have positive impacts on human wellbeing (Howell et al., 2008; LeBlanc et al., 2019). These activities not only benefit physical health, but they also help people relax and maintain a good mood. Meanwhile, some self-care methods are unhealthy, such as smoking and drinking alcohol. Nicotine in cigarettes has the effect of temporarily reducing stress (Kassel et al., 2003; Kober et al., 2017). However, in the long term, this is detrimental to health and can cause various lung and cardiovascular diseases. Alcoholic drinks, when consumed in small amounts, can have positive effects on health and could help energize people. However, excessive intake levels can cause severe damage to the internal organs in the long term; the effects could even be fatal (Blaine and Sinha, 2017). Although the negative side effects of smoking and drinking alcohol are widely recognized, such habits are still practiced by many to obtain instantaneous relief.

A study examining the relationship between entrepreneurial wellbeing and self-care behaviour patterns has been conducted in Australia. In the study, respondents were recruited from an Australian online entrepreneur group, meetup.com, and RMIT business school alumni. The results revealed that wellbeing levels are generally higher for established entrepreneurs than for nascent entrepreneurs. Moreover, established entrepreneurs are more likely to select healthy self-care methods while nascent entrepreneurs tend to choose unhealthy behaviours to relieve their stress (Wiklund et al., 2018). One possible reason for this phenomenon is that nascent entrepreneurs may be more stressed as they have just entered a highly uncertain environment that involves major financial burdens and job insecurity. Therefore, nascent entrepreneurs may prefer smoking and drinking, which could provide them with swift yet short-term comfort, to healthy self-care behaviours, whose benefits are only evident in the long term.

Specific attention has focused on entrepreneurs with ADHD and dyslexia. Previous research has found that people with ADHD or dyslexia tend to engage more in negative self-care behaviours, such as smoking and drinking alcohol, and are more likely to suffer from sleeping problems (Kooij et al., 2019). The Australian study also found that those with ADHD or dyslexia were generally less healthy than those without, across all the self-care behaviours examined, with the only exception being meditation/yoga. While self-care is beneficial to all, it was found that those with mental health problems benefitted more from positive self-care behaviours than those without (Wiklund et al., 2018).

## Research Methodology

Two sets of survey questions were uploaded onto a professional online survey platform, Qualtrics, in Singapore. As the target study sample was entrepreneurs in Singapore, this platform was easier for the target sample to access. Two types of respondents were included in the study sample, namely established entrepreneurs and nascent entrepreneurs. Established entrepreneurs were defined as people who had already developed and operated their own businesses, while nascent entrepreneurs were defined as people who were still preparing to start their own businesses.

### Demographics

Participants for this study were recruited in three main ways. The first major source of participants was MBA students or alumni from the Business School of Singapore Management University, the National University of Singapore and Nanyang Technological University. Candidates are required to have at least 3 years' work experience to be able to apply for this MBA programme, and a significant proportion of students on this programme had already owned and operated their own businesses. The second major source was the undergraduate innovation and entrepreneurship clubs in these same universities. Members of these clubs are current undergraduates or graduates, either preparing to start their own businesses or having just started their own businesses. Lastly, the survey links were also posted on various online social media platforms to allow other relevant people who were interested in this study to complete the surveys.

The surveys were completely voluntary. The two sets of survey questions were customized to target the two different respondent groups. Most questions were identical, while several questions were set differently for different groups, considering that the established and nascent entrepreneurs are at completely different stages of their businesses. A total of 1,294 responses were collected within 1 month, with 710 responses from established entrepreneurs and 584 from nascent entrepreneurs. Among these responses, 700 from established entrepreneurs and 576 from nascent entrepreneurs were counted as effective, as they were sufficiently complete for statistical analysis. More specifically, only 346 responses from established entrepreneurs and 488 responses from nascent entrepreneurs were used for analysis in this study as only businesses established after 2012 were selected for the study. The age range of the businesses owned by the respondents was very broad, with many even being established before 2000. This study focused mainly on recent phenomena and behaviours; hence, only businesses that had been established for <7 years were selected.

Both groups were asked to provide some basic personal information, including their age, gender, education level and marital status. They were also asked about their years of experience in the relevant industry, whether they had previous experience of starting a new business and whether their parents had their own businesses. In total, the average age for the whole sample was 33.3; specifically, the number was 37.1% for the established group and 30.6% for the nascent group. The result was within expectations, as the nascent entrepreneurs consisted of more young people, while the established group contained a more experienced and older generation of entrepreneurs. In this study sample, 66% were male and 29% were female; the remaining 5% did not wish to disclose their gender. Specifically, for the established entrepreneurs, 61% were male and 35% were female; for the nascent entrepreneurs, 70% were male and 24% were female. The gender composition for the two categories was considerably different, with the percentage of males significantly higher than that of females in both categories. It was noted that the percentage of females in the nascent group was much lower than that in the established group, possibly because of the far more stressful and difficult macro-environment in Singapore recently. As women are typically less likely to venture into risky and uncertain activities in particularly difficult times, they may be even more likely to avoid starting new ventures.

### Measurements

Questions were set in both surveys to test respondents' ADHD symptoms, dyslexia symptoms, self-care behaviour and overall life satisfaction levels. In this project, the adult ADHD self-report scale (ASRS-6) was used as a screening test to identify respondents who potentially have ADHD. It was adopted from Kessler et al. (2005) and this scale is widely used by scholars when measure ADHD (Kessler et al., 2007; Hines et al., 2012; Stanton et al., 2018). There are six questions, measured on a five-point Likert scale, in which 1 = Never, 2 = Rarely, 3 = Sometimes, 4 = Often and 5 = Very Often. Based on these screening rules, respondents were further categorized into two groups, one consisting of those with very high potential to have ADHD and the other consisting of the remainder, who were considered to have no ADHD symptoms.

The Adult Dyslexia Checklist proposed by Vinegard (1994) was used to identify those that may potentially have dyslexia. A total of 14 questions were asked, with 0 = answer “No” and 1 = answer “Yes”. Respondents who had a score equal to or above four would be considered to potentially have dyslexia. To measure the participants' wellbeing levels, a question was set that required participants to rate their overall satisfaction levels with life. The question consisted of five sub-questions, measured on a seven-point scale, with 1 = strongly disagree and 7 = strongly agree. The total score for each response was tallied. The neutral point was 20: scores below 20 were considered poor in terms of wellbeing and the rest were considered good (Diener et al., 1985).

For self-care behaviours, respondents were asked to select how frequently they had performed each activity/item in a week, with 1 = Never, 2 = 1 time, 3 = 2 to 3 times, 4 = 4 to 5 times and 5 = 6 to 7 times. Some self-care behaviours were considered healthy, including getting enough sleep, exercise, yoga and meditation, eating healthy food and finding comfort in religion; some were considered unhealthy, such as smoking and drinking alcohol (Cook-Cottone and Guyker, 2018).

Other questions were asked regarding the general information about the businesses owned by the entrepreneurs, including the business location, industry type, description of the products or services offered by the business, estimated yearly revenue or profits, average working time on the business, number of employees in the business and many others. Though not discussed in this report, these questions could serve as filters or evidence for the selection of effective responses. The answers and results could also be used for future studies and other research directions.

## Results and Discussion

### ADHD, Dyslexia, and Entrepreneurs' Wellbeing

**Figure 1:**
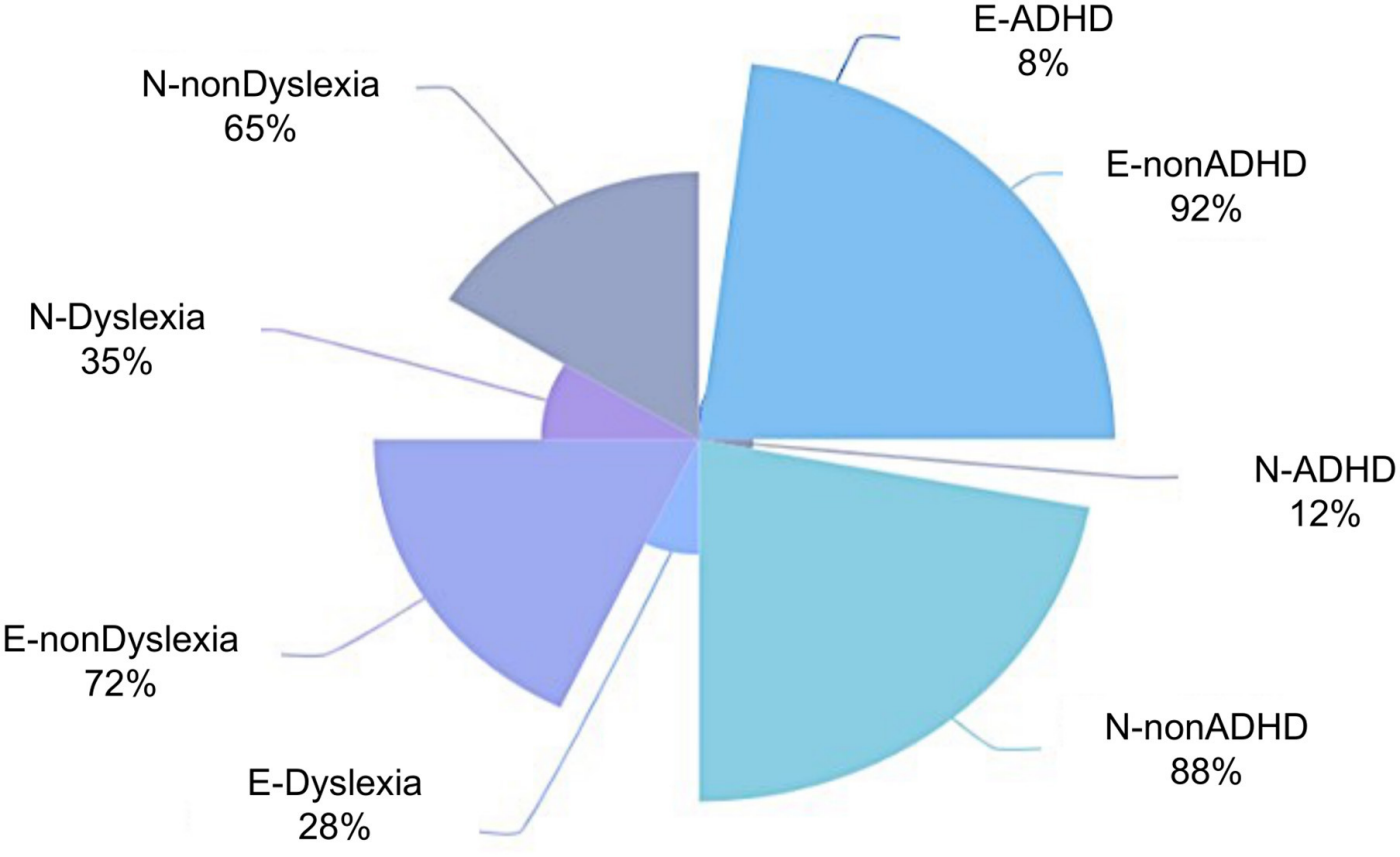
ADHD and Dyslexia distribution among two groups.

As showed in Figure 1, the percentage of ADHD was 8% for the established group and 12% for the nascent group, which are fairly similar, with the percentage for the nascent entrepreneurs being slightly higher. For dyslexia symptoms, compared to ADHD, the numbers were much higher for both groups, with 28%, for the established group; and 35%, for the nascent group. The percentage of dyslexia for the nascent group was slightly higher than that for the established group.

It was observed that the percentages of both people having ADHD symptoms and people having dyslexia symptoms were higher for the nascent group than for the established group. This might be explained by the different stress levels experienced by the two groups. The higher stress level faced by nascent entrepreneurs may be more likely to induce ADHD or dyslexia behaviours.

**Figure 2:**
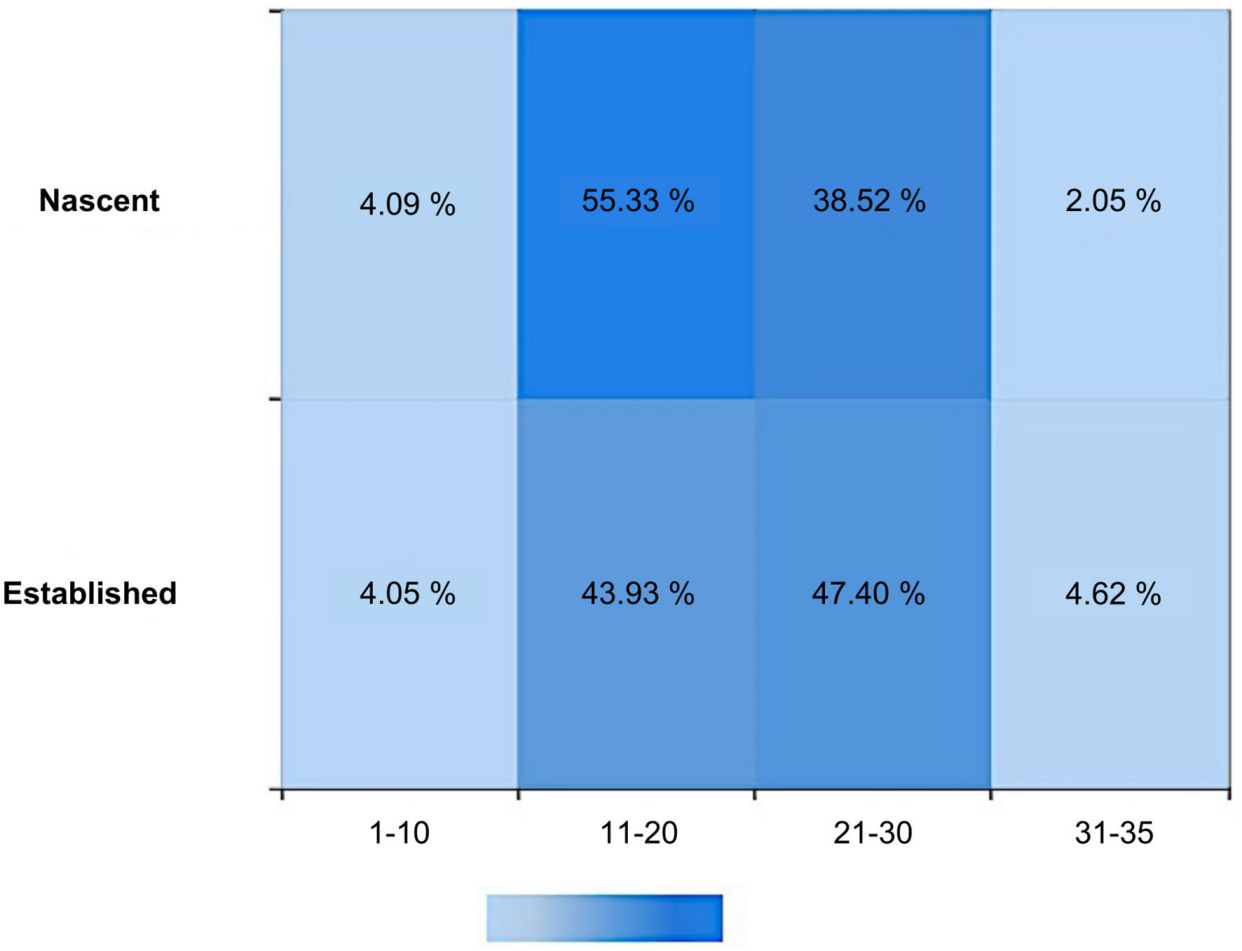
Wellbeing score distribution of established and nascent entrepreneurs.

For the well-being score (Figure 2), the average score of the established entrepreneurs was 21.618. A total of 90 respondents scored above the 20-point line, which was 52% of the total number of established entrepreneurs. For the nascent entrepreneurs, the average score was 20.5, lower than that for established entrepreneurs. In the nascent group, 198 out of 488 achieved a score above 20, which amounted to only 41% of the total number of nascent entrepreneurs. Both the average score and the percentage of people with good overall wellbeing among the nascent entrepreneurs were far lower than the equivalent values among established entrepreneurs. This suggests that established entrepreneurs generally have a higher level of satisfaction with their lives, possibly because they have already passed the most uncertain and difficult period of starting a business and are now enjoying the benefits and stable income resulting from having their own businesses. However, as nascent entrepreneurs are still at the very first stage of starting new businesses, they encounter a greater amount of stress and uncertainty and may have to spend far more time working than regular office workers. Moreover, their financial burdens are expected to be very high, as a considerable amount of money has to be invested to initiate their operations before any profits could be realized by the business.

**Figure 3:**
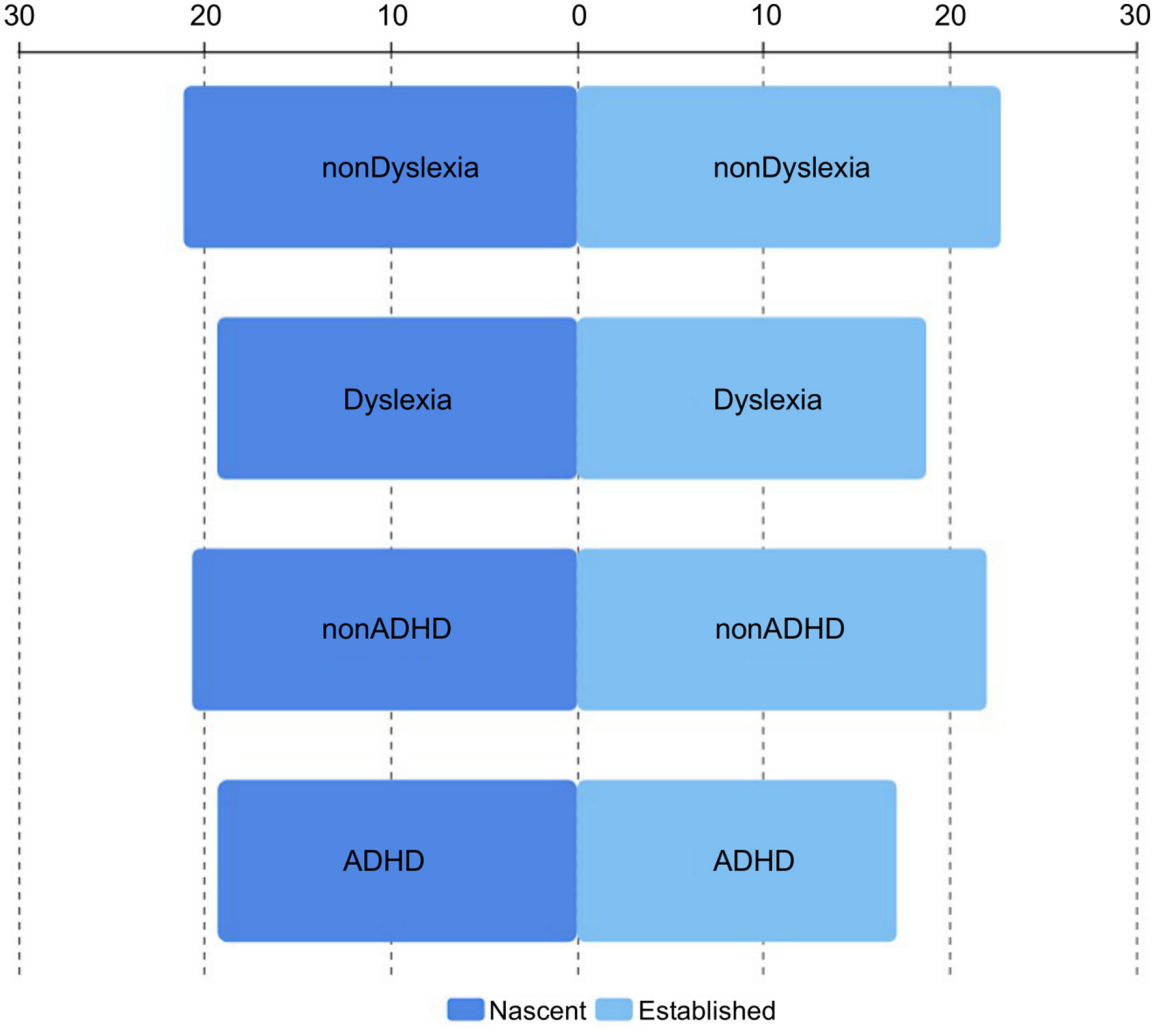
Comparison of wellbeing scores of different entrepreneurs' groups.

Next, the wellbeing level of those with ADHD symptoms was examined. As indicated in Figure 3, the average score for established entrepreneurs with ADHD symptoms was 17.15, significantly lower than the score of 21.98 found in those without ADHD symptoms. For nascent entrepreneurs, the average score for those with ADHD symptoms was 19.3, which is also lower than the score for those without ADHD symptoms. However, the difference is minor, possibly because nascent entrepreneurs generally all suffer from very high levels of stress, as well as mental and physical fatigue. Thus, their wellbeing levels all tended to be low and the difference between ADHD and non-ADHD became unclear. In terms of dyslexia, the average well-being score for the nascent group was 19.3, much lower than the score of 21.1 found in the nascent non-dyslexia group. The number for the established dyslexia group was even lower, 18.7, while the established non-dyslexia group scored fairly high, 22.7.

**Figure 4:**
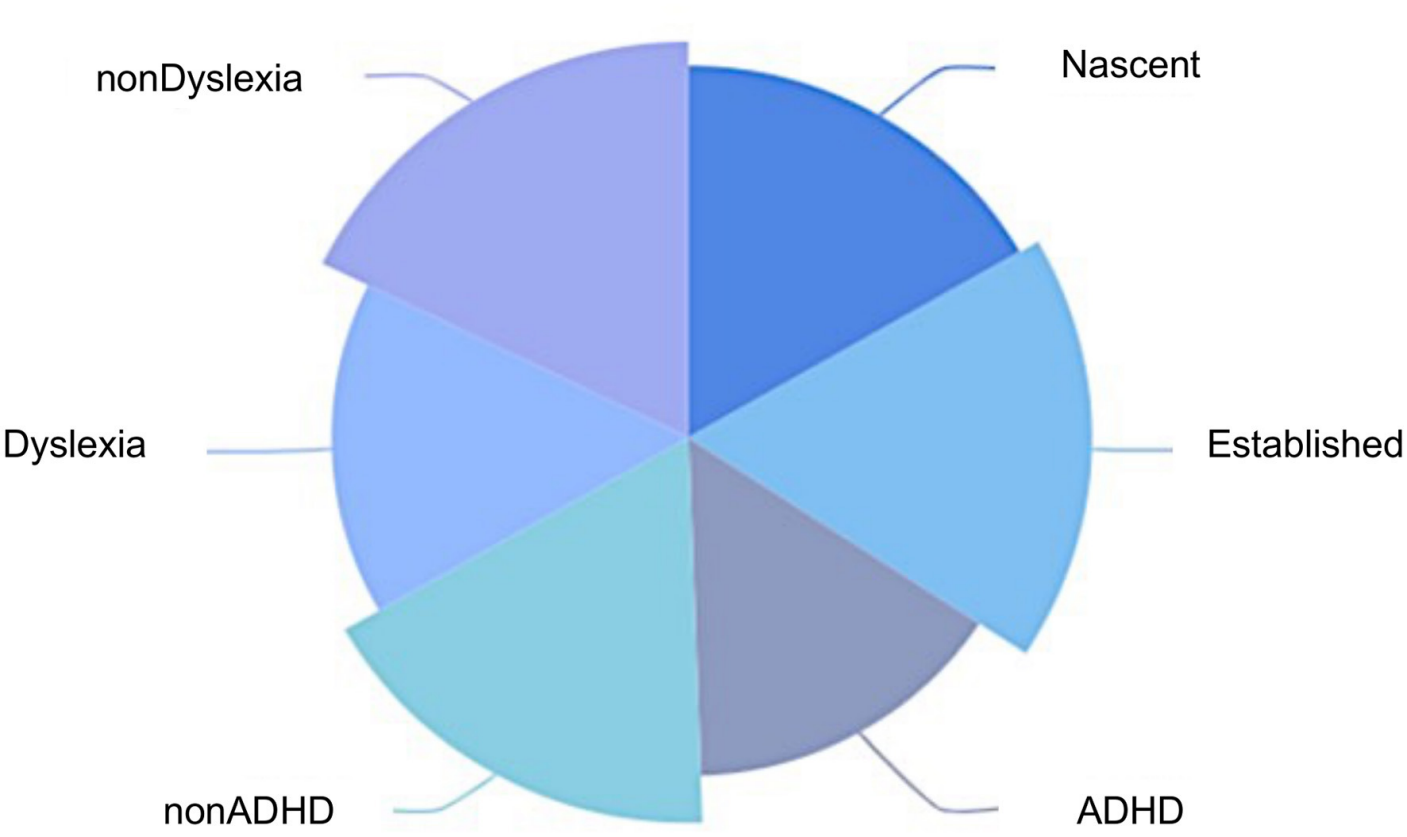
Comparison of wellbeing score of different groups of entrepreneurs.

Overall, as vividly shown in the Figure 4, the wellbeing levels of those with ADHD or dyslexia symptoms were much lower than those without. This is consistent with the findings of previous psychological research, which have reported that mental health problems have negative influences on life satisfaction levels (Fergusson et al., 2015). Furthermore, the results closely resembled research recent findings from Australia (Wiklund et al., 2018).

### Wellbeing and Self Care Behaviours

In this section, the relationship between self-care behaviours and entrepreneurial wellbeing is examined, with a specific focus on those with mental disorders such as ADHD and dyslexia. The importance of self-care to mental health and wellbeing has been proven by research. High quality sleep, regular exercise and healthy food not only benefit physical health, but are also important to mental health (Howell and Howell, 2008; Howell et al., 2008). In this study, the seven most commonly practiced self-care behaviours were identified and examined. Respondents were asked how often they practiced each item on a weekly basis. Different cut-offs were set for different items where appropriate. Based on the cut-offs, the percentage of people engaged at high frequency levels in each item was calculated and a comparison was made between different categories of entrepreneurs. As shown in Table 1, it was seen that nascent entrepreneurs were more likely to be engaged in self-care behaviours than established ones. More alcohol drinking was found in established entrepreneurs. With 69.36%, while more smoke behaviours were appeared in nascent ones (84.62).

**Table 1:** Percentage engaged at high frequency levels for different groups of entrepreneurs.

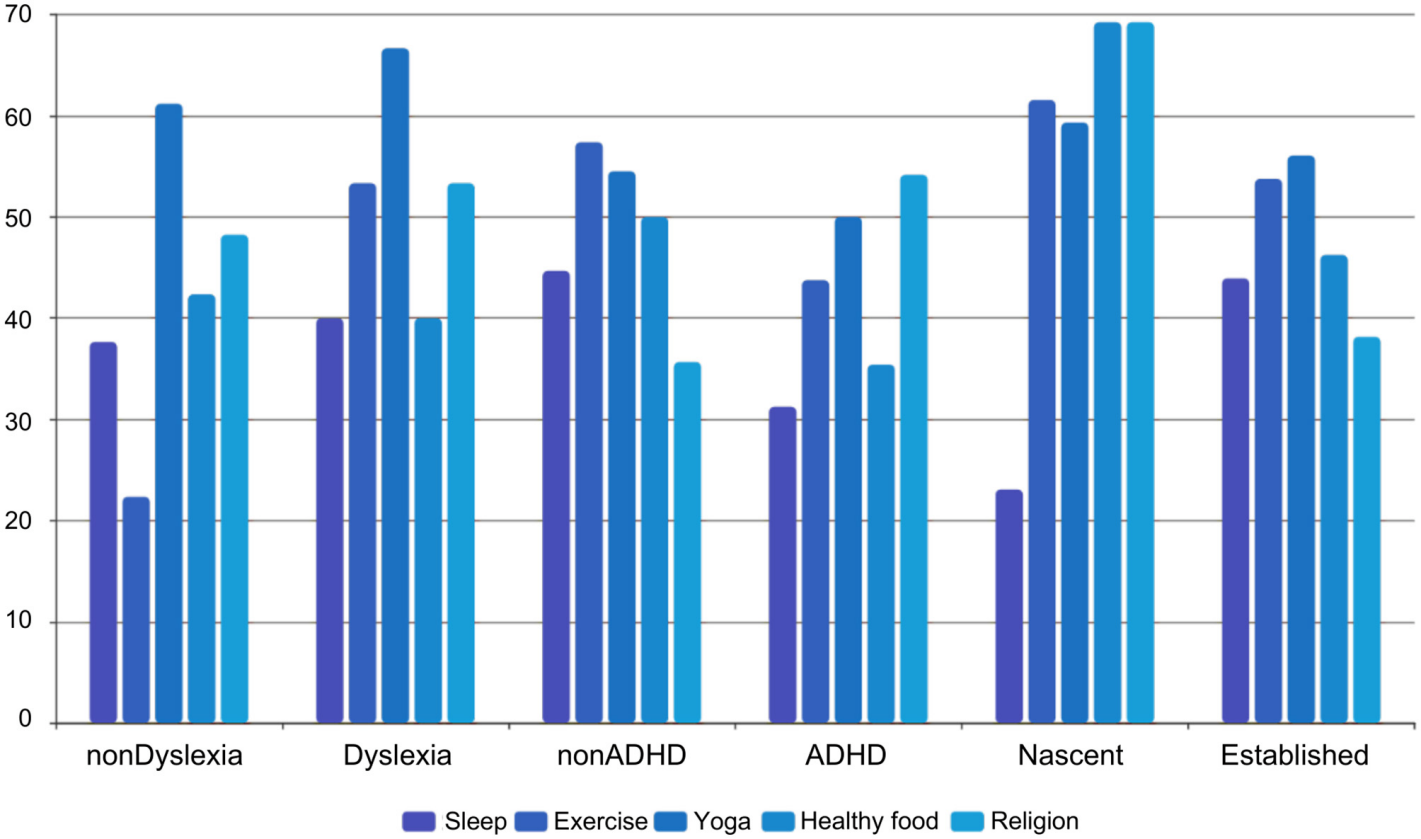							
	**Sleep**	**Exercise**	**Yoga**	**Healthy food**	**Religion**	**Alcohol**	**Smoke**
NonDyslexia	37.65	22.35	61.18	42.35	48.24	32.94	41.18
Dyslexia	40.00	53.33	66.67	40.00	53.33	23.33	43.33
NonADHD	44.67	57.38	54.51	50.00	35.66	22.95	30.74
ADHD	31.25	43.75	50.00	35.42	54.17	37.50	60.42
Nascent	23.08	61.54	59.32	69.23	69.23	61.54	84.62
Established	43.93	53.76	56.07	46.24	38.15	59.36	64.16

For the four healthy self-care items, the percentage of people engaged at high frequency levels was almost the same between the two groups of entrepreneurs for each individual item examined. This observation is reasonable, as entrepreneurs enjoy a high level of autonomy and flexibility to arrange their time and work, so it is easier for them to make time for healthy behaviours such as exercise and meditation. However, for the two unhealthy items, smoking and drinking, it was found that the established group smoked and drank far more than the nascent group. This result differs considerably from the findings of previous studies in western societies, in which nascent entrepreneurs were found to engage in healthy behaviours far less than established entrepreneurs. Meanwhile, in the latter studies, smoking and drinking alcohol were far more common among nascent entrepreneurs than among established entrepreneurs due to the higher levels of stress and work commitments experienced by the former. This difference might be explained by the different cultures in Chinese society and western societies. Singapore is well known for its drinking culture, especially in the areas of business negotiation and relationship building. Established entrepreneurs in Singapore usually attend many dining and entertainment occasions every week, at which they are expected to smoke and drink a considerable amount.

Contrary to general expectations, the younger generation in Singapore now places far more emphasis on, and effort into, promoting their health and preserving youth, compared to the older generation. As the nascent entrepreneurs examined in this study were dominated by the younger generation (aged between 20 and 30), it could be expected that this group of young entrepreneurs in Singapore would refrain from smoking and drinking in order to maintain a relatively healthier lifestyle.

Next, a comparison was made between entrepreneurs with symptoms of mental health problems and those without. Generally, entrepreneurs with ADHD symptoms get enough sleep less frequently than those without ADHD. It was unexpected that the results for exercise and healthy food were almost identical for the two groups, while those with ADHD actually engaged in yoga, meditation and religious behaviours more often than those without ADHD. Concerning the two negative behaviours, those with ADHD displayed a significantly higher frequency of smoking and drinking compared to those without, and the same pattern was also observed for dyslexia. This corresponded to other research findings, which have reported that people with ADHD tend to have more problems sleeping and are more likely to smoke and drink a lot (Ohlmeier et al., 2008). However, in terms of healthy self-care behaviours, the dyslexia group exhibited quite different patterns from the ADHD group, except for sleep. Those with dyslexia symptoms exercised and ate healthy food much less than those without. This is consistent with previous research findings that report how people with dyslexia tend to take less care of themselves and live a less healthy lifestyle (Germanò et al., 2010; Lonergan et al., 2019). The patterns discussed above might explain the lower well-being level experienced by entrepreneurs with ADHD or dyslexia symptoms.

## Discussion and implications

This study found that within the sample of established and nascent Singaporean entrepreneurs, 10.3% were highly likely to have ADHD and 31.9% may have dyslexia. The rate is far higher than that for the normal adult population, among whom 5% are likely to have ADHD (“ADHD Statistics”, 2019) and 15–20% are likely to have dyslexia (Adlof and Hogan, 2018; Lopes et al., 2020). This suggests a link between entrepreneurship and ADHD and dyslexia behaviours, which is consistent with previous research, such as that of Verheul et al. (2015) and Lerner et al. (2019). In general, established entrepreneurs have a higher level of life satisfaction than nascent entrepreneurs. This can be explained by nascent entrepreneurs suffering far more from stress and workload than established entrepreneurs. The latter have already reached the stage at which they can enjoy the benefits of being an entrepreneur, while nascent entrepreneurs have just started their ventures. Though they have a lower level of wellbeing, nascent entrepreneurs appear to be healthier than established entrepreneurs in terms of self-care behaviours. Compared with established entrepreneurs, nascent entrepreneurs show a significantly lower percentage in terms of engaging in highly frequent smoking or drinking alcohol. This result differs considerably from the results found in western societies. Specific attention focused on the group of entrepreneurs with ADHD or dyslexia symptoms. Overall, the wellbeing levels of those with ADHD or dyslexia symptoms are far lower than those without. This is consistent with previous psychological research findings, that mental health problems have negative influences on life satisfaction levels (Fergusson et al., 2015). It was also observed that those with ADHD or dyslexia symptoms practiced a less healthy lifestyle than those without, which might be one reason why they experience lower levels of wellbeing.

There are some implications of this study. Firstly, the results highlight the levels of mental health and wellbeing in entrepreneur groups. Due to the uncertainty and high risk of this industry, entrepreneurs are more likely to contract ADHD or dyslexia symptoms. Secondly, the relatively low level of wellbeing among nascent entrepreneurs requires the relevant regulatory bodies or organizations to provide more welfare to support those less experienced in business. Thirdly, the results indicate that veteran entrepreneurs appear to practice more unhealthy behaviours, like smoking or alcohol, so they should be reminded to undertake self-care behaviours.

## Limitations and future directions

Although this study revealed significant results, they must be considered alongside the limitations of the study. Multiple openings remain for further investigation in future. To begin with, the study was conducted in Singapore, so the diversity of demographics, different personalities and social backgrounds might have had an impact on the results. Hence, future studies should compare different research contexts. Next, potential self-report bias might exist in the survey. Respondents may have over- or underestimated their levels of mental disorder and wellbeing in their past communicative experiences. Future studies should thus use more objective measures, such as clinical assessments of mental and psychological status. Lastly, the samples were mainly drawn from alumni from the National University of Singapore, Singapore Management University and Nanyang Technological University, which raises homogeneity issues. Future studies should conduct research with a large and diverse sample.

## Conclusion

This study examined the mental health, self-care behaviours and wellbeing among veteran and nascent entrepreneurs in Singapore. The results showed various similarities, like the high frequency of ADHD or dyslexia symptoms that they display as part of their occupation among nascent and established entrepreneurs. It is true that in a fiercely competitive business world, it is more likely to contract ADHD for all entrepreneurs. However, when ADHD symptoms are considered in conjunction with entrepreneurial passion, it may be possible to understand how and why some entrepreneurs with ADHD symptoms overcome their disadvantages to manage successful companies (Hatak et al., 2021). It can also explain why this group has high rate of ADHD. Some differences between two entrepreneurial categories were also identified, such as their levels of wellbeing and awareness of self-care behaviours. To be more precise, nascent tend to be healthier in the lifestyle and they have more self-care intentions and behaviours compared. It explained by the different cultures and habits in Singaporean society. Since cultural is a potential factor that influences individual's self-care (Permana et al., 2019; Litam, 2020). This study advances understanding in the nuanced differences among two categories of entrepreneurs in Asian context.

## Data Availability Statement

The original contributions presented in the study are included in the article/Supplementary Material, further inquiries can be directed to the corresponding author.

## Ethics Statement

All procedures performed in studies involving human participants were in accordance with the Ethical Standards of the Institutional Research Committee (Universities Malaya) and with the 1964 Helsinki Declaration and its later amendments or comparable Ethical Standards. The Ethics Committee waived the requirement of written informed consent for participation.

## Author Contributions

JG and ZX designed the guideline of the articles. SW, PO, and ZX contributed to the drafting of material for individual section. MG compiled the writing and conducted the analysis. JG and SW aligned the paper. YL and PO reviewed and provided corrections on the original draft. All authors contributed to the article and approved the submitted version.

## Conflict of Interest

The authors declare that the research was conducted in the absence of any commercial or financial relationships that could be construed as a potential conflict of interest.

## Publisher's Note

All claims expressed in this article are solely those of the authors and do not necessarily represent those of their affiliated organizations, or those of the publisher, the editors and the reviewers. Any product that may be evaluated in this article, or claim that may be made by its manufacturer, is not guaranteed or endorsed by the publisher.

## References

[B1] Adlof S. M. Hogan T. P. (2018). Understanding dyslexia in the context of developmental language disorders. Lang. Speech Hear Serv. Sch. 49, 762–773. 10.1044/2018_LSHSS-DYSLC-18-0049 30458538 PMC6430503

[B2] Alexander-Passe N. (2015). Dyslexia and Mental Health: Helping people identify destructive behaviours and find positive ways to cope. Philadelphia, PA: Jessica Kingsley Publishers.

[B3] Archer D. (2015). The ADHD advantage: What you thought was a diagnosis may be your greatest strength. New York, NY: Penguin.

[B4] Baron R. A. Ensley M. D. (2006). Opportunity Recognition as the Detection of Meaningful Patterns: Evidence from Comparisons of Novice and Experienced Entrepreneurs. Management Sci. 52, 1331–1344. 10.1287/mnsc.1060.0538

[B5] Blaine S. K. Sinha R. (2017). Alcohol, stress, and glucocorticoids: From risk to dependence and relapse in alcohol use disorders. Neuropharmacology. 122, 136–147. 10.1016/j.neuropharm.2017.01.037 28159647 PMC5479733

[B6] Cook-Cottone C. P. Guyker W. M. (2018). The development and validation of the Mindful Self-Care Scale (MSCS): an assessment of practices that support positive embodiment. Mindfulness. 9, 161–175. 10.1007/s12671-017-0759-1

[B7] Davis J. C. Bryan S. Li L. C. Best J. R. Hsu C. L. Gomez C.. (2015). Mobility and cognition are associated with wellbeing and health related quality of life among older adults: a cross-sectional analysis of the vancouver falls prevention cohort. BMC Geriatr. 15, 1–7. 10.1186/s12877-015-0076-2 26142897 PMC4491415

[B8] Diener E. Emmons R. A. Larsen R. J. Griffin S. (1985). Satisfaction with life scale (SWLS). J. Pers. Assess. 49, 71–75. 10.1207/s15327752jpa4901_13 16367493

[B9] DuPaul G. J. Power T. J. Anastopoulos A. D. Reid R. (1998). ADHD Rating Scale—IV: Checklists, Norms, and Clinical Interpretation. New York, NY: Guilford press. 10.1037/t00680-000

[B10] Engel Y. van Werven R. Keizer A. (2020). How novice and experienced entrepreneurs name new ventures. J. Small Bus. Manag. 1–31. 10.1080/00472778.2020.1738820

[B11] Estrin S. Mickiewicz T. Stephan U. Wright M. (2019). Entrepreneurship in emerging markets. The Oxford handbook of management in emerging markets. p. 457. 10.1093/oxfordhb/9780190683948.013.21

[B12] Fergusson D. McLeod G. Horwood L. J. Swain N. Chapple S. Poulton R. (2015). Life satisfaction and mental health problems (18 to 35 years). Psychological Med. 45, 2427–2436. 10.1017/S0033291715000422 25804325

[B13] Fletcher J. M. (2014). The effects of childhood ADHD on adult labor market outcomes. Health Econom. 23, 159–181. 10.1002/hec.2907 23427026 PMC6714576

[B14] Germanò E. Gagliano A. Curatolo P. (2010). Comorbidity of ADHD and dyslexia. Dev Neuropsychol. 35, 475–493. 10.1080/87565641.2010.494748 20721770

[B15] Hakulinen C. Elovainio M. Arffman M. Lumme S. Pirkola S. Keskimäki I.. (2019). Mental disorders and long-term labour market outcomes: nationwide cohort study of 2 055 720 individuals. Acta Psychiatrica Scandinavica. 140, 371–381. 10.1111/acps.13067 31254386

[B16] Halunko V. Ivanyshchuk A. Popovych T. (2018). Global experience of social entrepreneurship development. Balt. J. Econ. Stud. 4, 62–67. 10.30525/2256-0742/2018-4-1-62-67

[B17] Hatak I. Chang M. Harms R. Wiklund J. (2020). ADHD symptoms, entrepreneurial passion, and entrepreneurial performance. Small Bus. Econ. 1–21.

[B18] Hatak I. Chang M. Harms R. Wiklund J. (2021). ADHD symptoms, entrepreneurial passion, and entrepreneurial performance. Small Bus. Econ. 57, 1693–1713. 10.1007/s11187-020-00397-x

[B19] Haver A. Olsen E. Akerjordet K. (2019). Well-being among hotel managers: A study on the influence of job stressors and cognitive reappraisal. Int. J. Contemp. Hosp. Manag. 31, 1819–1835. 10.1108/IJCHM-11-2017-0737

[B20] Hines J. L. King T. S. Curry W. J. (2012). The adult ADHD self-report scale for screening for adult attention deficit–hyperactivity disorder (ADHD). J. Am. Board Fam. Med. 25, 847–853. 10.3122/jabfm.2012.06.120065 23136325

[B21] Howell A. J. Digdon N. L. Buro K. Sheptycki A. R. (2008). Relations among mindfulness, well-being, and sleep. Pers. Individ. Differ. 45, 773–777. 10.1016/j.paid.2008.08.005

[B22] Howell R. T. Howell C. J. (2008). The relation of economic status to subjective well-being in developing countries: a meta-analysis. Psychol. Bull. 134, 536. 10.1037/0033-2909.134.4.536 18605819

[B23] Kassel J. D. Stroud L. R. Paronis C. A. (2003). Smoking, stress, and negative affect: correlation, causation, and context across stages of smoking. Psychol. Bull. 129, 270. 10.1037/0033-2909.129.2.270 12696841

[B24] Kessler R. C. Adler L. Ames M. Demler O. Faraone S. Hiripi E.. (2005). The World Health Organization Adult ADHD Self-Report Scale (ASRS): a short screening scale for use in the general population. Psychol. Med. 35, 245–256. 10.1017/S0033291704002892 15841682

[B25] Kessler R. C. Adler L. A. Gruber M. J. Sarawate C. A. Spencer T. Van Brunt D. L. (2007). Validity of the World Health Organization Adult ADHD Self-Report Scale (ASRS) Screener in a representative sample of health plan members. Int. J. Methods Psychiatr. Res. 16, 52–65. 10.1002/mpr.208 17623385 PMC2044504

[B26] Kober H. Brewer J. A. Height K. L. Sinha R. (2017). Neural stress reactivity relates to smoking outcomes and differentiates between mindfulness and cognitive-behavioral treatments. Neuroimage. 151, 4–13. 10.1016/j.neuroimage.2016.09.042 27693614 PMC5373945

[B27] Kooij J. Bijlenga D. Salerno L. Jaeschke R. Bitter I. Balazs J.. (2019). Updated European Consensus Statement on diagnosis and treatment of adult ADHD. Eur. Psychiat. 56, 14–34. 10.1016/j.eurpsy.2018.11.001 30453134

[B28] LeBlanc S. Uzun B. Aydemir A. (2019). Structural relationship among mindfulness, reappraisal and life satisfaction: The mediating role of positive affect. Current Psychol. 1–10. 10.1007/s12144-019-00383-x

[B29] Lerner D. A. Hunt R. A. Verheul I. (2018). Dueling banjos: Harmony and discord between ADHD and entrepreneurship. Acad. Manag. Perspect. 32, 266–286. 10.5465/amp.2016.0178

[B30] Lerner D. A. Verheul I. Thurik R. (2019). Entrepreneurship and attention deficit/hyperactivity disorder: a large-scale study involving the clinical condition of ADHD. Small Bus. Econ. 53, 381–392. 10.1007/s11187-018-0061-1

[B31] Litam S. D. A. (2020). “Take Your Kung-Flu Back to Wuhan”: counseling Asians, Asian Americans, and Pacific Islanders with race-based trauma related to COVID-19. Professional Counselor. 10, 144–156. 10.15241/sdal.10.2.144

[B32] Lonergan A. Doyle C. Cassidy C. MacSweeney Mahon S. Roche R. A. Boran L.. (2019). A meta-analysis of executive functioning in dyslexia with consideration of the impact of comorbid ADHD. J Cognit. Psychol. 31, 725–749. 10.1080/20445911.2019.1669609

[B33] Lopes J. A. Gomes C. Oliveira C. R. Elliott J. G. (2020). Research studies on dyslexia: Participant inclusion and exclusion criteria. Eur. J. Spec. Needs Educ. 35, 587–602. 10.1080/08856257.2020.1732108

[B34] Lyon G. R. Shaywitz S. E. Shaywitz B. A. (2003). A definition of dyslexia. Ann Dyslexia. 53, 1–14. 10.1007/s11881-003-0001-9

[B35] McGowan P. van der Sijde P. Kirby D. (2008). The role of universities in the entrepreneurship industry: Promoting the entrepreneurship agenda in HEIs. Ind. High. Educ. 22, 49–59. 10.5367/000000008783876986

[B36] Mensmann M. Frese M. (2019). Who stays proactive after entrepreneurship training? Need for cognition, personal initiative maintenance, and well-being. J. Organ. Behav. 40, 20–37. 10.1002/job.2333

[B37] Moore C. B. McIntyre N. H. Lanivich S. E. (2021). ADHD-related neurodiversity and the entrepreneurial mindset. Entrep. Theory Pract. 45, 64–91. 10.1177/1042258719890986

[B38] Nordqvist C. (2017). Mental health: Definition, common disorders, and early signs. Brighton: Medical News Today.

[B39] Ohlmeier M. D. Peters K. Wildt B. T. T. Zedler M. Ziegenbein M. Wiese B.. (2008). Comorbidity of alcohol and substance dependence with attention-deficit/hyperactivity disorder (ADHD). Alcohol & Alcoholism. 43, 300–304. 10.1093/alcalc/agn014 18326548

[B40] Permana I. Ormandy P. Ahmed A. (2019). Maintaining harmony: how religion and culture are interwoven in managing daily diabetes self-care. J. Relig. Health. 58, 1415–1428. 10.1007/s10943-019-00819-5 31011937

[B41] Stanton K. Forbes M. K. Zimmerman M. (2018). Distinct dimensions defining the Adult ADHD Self-Report Scale: implications for assessing inattentive and hyperactive/impulsive symptoms. Psychol. Assess. 30, 1549. 10.1037/pas0000604 29878817

[B42] Thurik R. Khedhaouria A. Torres O. Verheul I. (2020). ADHD symptoms and entrepreneurial orientation of small firm owners. In: Wiley 111 River St. NJ USA: Hoboken 07030-5774. p 568. 10.1111/apps.12062

[B43] Tsuchiya M. Kawakami N. Ono Y. Nakane Y. Nakamura Y. Fukao A.. (2012). Impact of mental disorders on work performance in a community sample of workers in Japan: the World Mental Health Japan Survey 2002–2005. Psychiatry Res. 198, 140–145. 10.1016/j.psychres.2011.10.014 22374551

[B44] Tucker R. Zuo L. Marino L. D. Lowman G. H. Sleptsov A. (2021). ADHD and entrepreneurship: Beyond person-entrepreneurship fit. J. Bus. Ventur. Insights. 15, e00219. 10.1016/j.jbvi.2020.e00219

[B45] Ucbasaran D. Wright M. Westhead P. Busenitz L. W. (2003). “The impact of entrepreneurial experience on opportunity identification and exploitation: habitual and novice entrepreneurs”, In Cognitive Approaches to Entrepreneurship Research (Vol. 6, pp. 231-263). Katz J. A. Shepherd D. A. (Eds.), Emerald Group Publishing Limited. 10.1016/S1074-7540(03)06008-2

[B46] Verheul I. Block J. Burmeister-Lamp K. Thurik R. Tiemeier H. Turturea R. (2015). ADHD-like behavior and entrepreneurial intentions. Small Bus. Econ. 45, 85–101. 10.1007/s11187-015-9642-4

[B47] Vinegard M. (1994). A revised adult dyslexia check list. EDUCARE-LONDON-NATIONAL BUREAU FOR HANDICAPPED STUDENTS- 21–21.

[B48] Westhead P. Ucbasaran D. Wright M. (2005). Experience and cognition: do novice, serial and portfolio entrepreneurs differ? Int Small Bus J. 23, 72–98. 10.1177/0266242605049104

[B49] Wienen A. W. Sluiter M. N. Thoutenhoofd E. de Jonge P. Batstra L. (2019). The advantages of an ADHD classification from the perspective of teachers. Eur. J. Spec. Needs Educ. 34, 649–662. 10.1080/08856257.2019.1580838

[B50] Wiklund J. Hatak I. Patzelt H. Shepherd D. A. (2018). Mental disorders in the entrepreneurship context: When being different can be an advantage. Acad Manag Perspect. 182–206. 10.5465/amp.2017.0063

[B51] Wiklund J. Lomberg C. Alkærsig L. Miller D. (2019). When ADHD Helps and Harms in Entrepreneurship: An Epidemiological Approach. Paper presented at the Academy of Management Proceedings. 10.5465/AMBPP.2019.17481abstract

[B52] Wiklund J. Patzelt H. Dimov D. (2016). Entrepreneurship and psychological disorders: How ADHD can be productively harnessed. J. Bus. Ventur. Insights. 6, 14–20. 10.1016/j.jbvi.2016.07.001

[B53] Wiklund J. Yu W. Tucker R. Marino L. D. (2017). ADHD, impulsivity and entrepreneurship. J. Bus. Ventur. 32, 627–656. 10.1016/j.jbusvent.2017.07.002

[B54] World Health Organization. (2000). Mental Health and Work: Impact, Issues and Good Practices. Available online at: https://www.who.int/mental_health/media/en/712.pdf

