# Supplementary material for: Established and Nascent Entrepreneurs: Comparing the Mental Health, Self-Care Behaviours and Wellbeing in Singapore
Source: Front Sociol. 2022 Mar 25;7:843101. doi: 10.3389/fsoc.2022.843101 (PMC8990876; doi:10.3389/fsoc.2022.843101)
Supplement: Supplementary file 1 [file Table_1.DOCX]

Supporting information (not for publish)


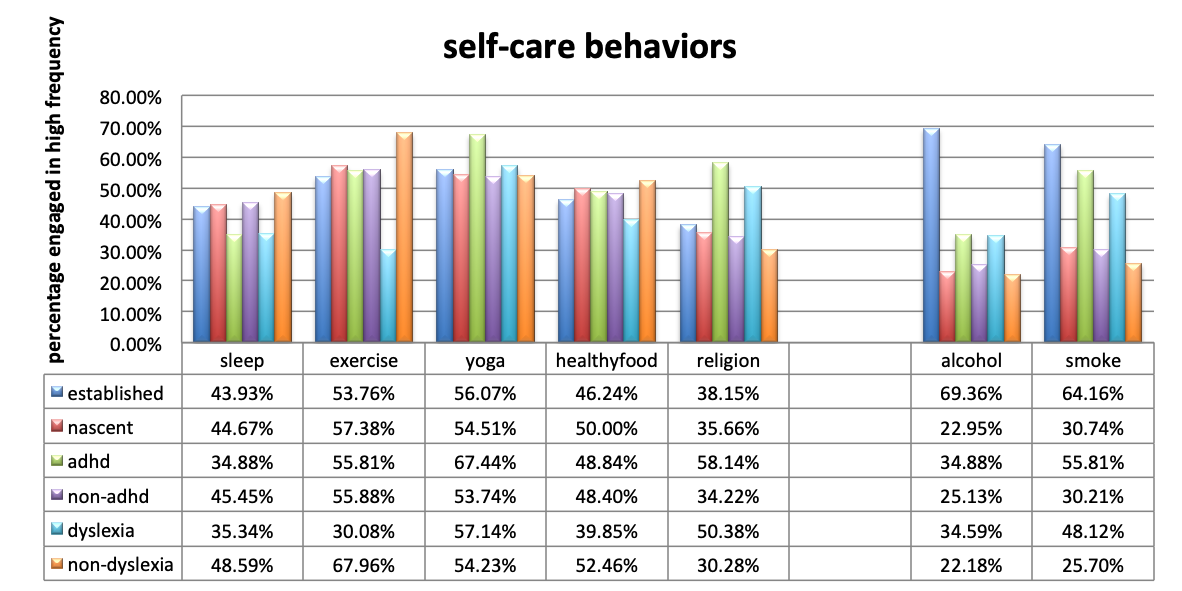


Figure S1. Percentage engaged in high frequency for different groups of entrepreneurs.


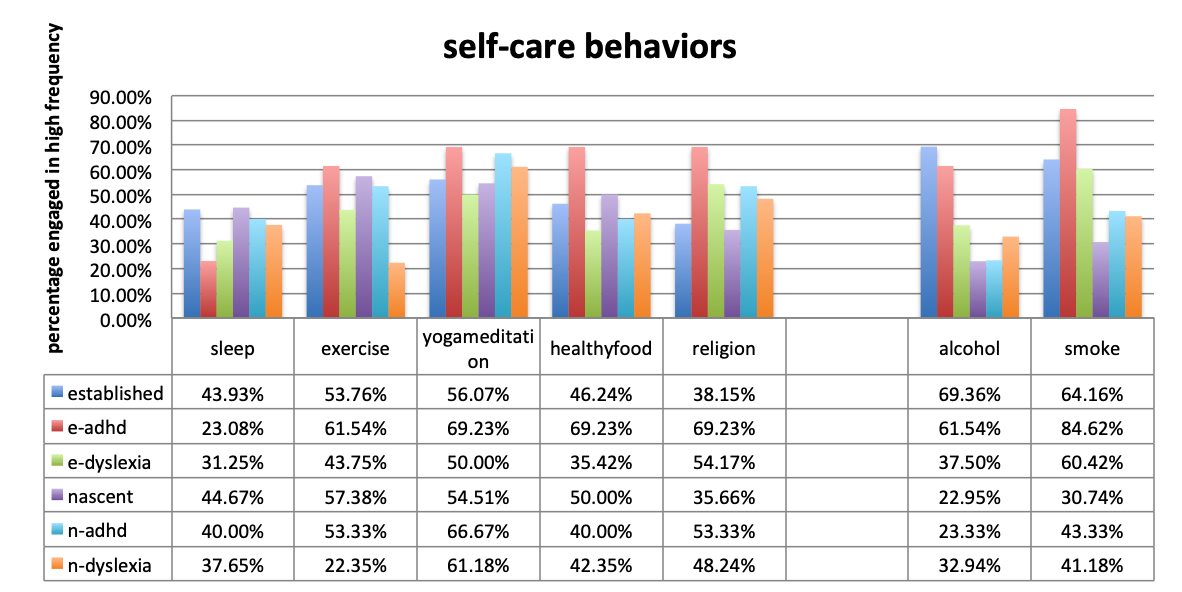


Figure S2. Percentage engaged in high frequency for different groups of entrepreneurs.

## **Appendices**

1. **Similarities between ADHD Symptoms and Entrepreneurial Behaviours**

| **ADHD** | **ENTREPRENEUR** |
| --- | --- |
| Easily distracted - seems to always have something new to think about | Short attention span – constantly has new ideas for how to improve the business |
| Day dreamer – visual thinker | Strong visualisation of their goals, enabling the skill of painting pictures for others |
| Disorganised | Ability to change direction quickly |
| Socially undeveloped | Willingness to make mistakes to progress |
| Starts several projects at the same time, may not complete any of them | Flexible – approaches problems from several different angles, always ready to change direction if that is what is needed |
| Impulsive | Quick reaction to opportunities |
| Prone to act without taking the consequences into account | Impatience to grow their business |
| Low self esteem | Modesty about their achievements |
| Hyperactive | High energy levels and ability to sustain their drive to get results – always on the go |
| Inattention | Underestimation of how long it takes to achieve a goal |
| Not good at using words, written or spoken | Belief in hands on, practical experience rather than training |
| Distorted sense of time – e.g. will spend hours playing a video game without realising how much time has passed | Immerses themselves in the job and often does not realise how much time has passed |
| Intrudes | Strong focus on customers and their needs |
| Hands-on-learner | Hands-on-manager |

*Source: O’Connor 2002.

1. **Cut-offs for Self-Care Behaviours**

| Item | Low Frequency | High Frequency |
| --- | --- | --- |
| Enough sleep | $<$ 4 days (1, 2, 3) | $\geq$ 4 days (4, 5) |
| Exercise more than 30 mins | $<$ 2 days (1, 2) | $\geq$ 2 days (3, 4, 5) |
| Yoga/meditation | 0 day (1) | $\geq$ 1 day (2, 3, 4, 5) |
| Religion | 0 day (1) | $\geq$ 1 day (2, 3, 4, 5) |
| Eat healthy food | $<$ 4 days (1, 2, 3) | $\geq$ 4 days (4, 5) |
| Smoke (unhealthy) | 0 day (1) | $\geq$ 1 day (2, 3, 4, 5) |
| Drink alcohols (unhealthy) | $<$ 2 days (1, 2) | $\geq$ 2 days (3, 4, 5) |

1. **Selected Survey Questions**
2. **Dyslexia**

Please answer the following questions.

|  | No | Yes |
| --- | --- | --- |
| Do you find difficulty in telling left from right? | 🔿 | 🔿 |
| Is map reading or finding your way to a strange place confusing? | 🔿 | 🔿 |
| Do you take longer than you should to read a page of a book? | 🔿 | 🔿 |
| Do you find it difficult to remember the meaning of what you have read? | 🔿 | 🔿 |
| Do you dislike reading long books? | 🔿 | 🔿 |
| Is your spelling poor? | 🔿 | 🔿 |
| Is your handwriting difficult to read? | 🔿 | 🔿 |
| Do you find it difficult to take messages on the telephone and pass them on correctly? | 🔿 | 🔿 |
| Do you find it difficult to do sums in your head without using your fingers or paper? | 🔿 | 🔿 |
| When using the telephone, do you tend to get the numbers mixed up when you dial? | 🔿 | 🔿 |
| Do you mix up dates and times and miss appointments? | 🔿 | 🔿 |
| Do you find forms difficult and confusing? | 🔿 | 🔿 |
| Do you mix up bus numbers like 95 and 59? | 🔿 | 🔿 |
| Did you find it hard to learn your multiplication tables at school? | 🔿 | 🔿 |

1. **ADHD**

Please answer these questions based on how often you have had these experiences during the past 6 months.

|  | Never | Rarely | Someti-mes | Often | Very often |
| --- | --- | --- | --- | --- | --- |
| Trouble wrapping up the final details of a project, once the challenging parts have been done. | 🔿 | 🔿 | 🔿 | 🔿 | 🔿 |
| Difficulty getting things in order when you have to do a task that requires organization. | 🔿 | 🔿 | 🔿 | 🔿 | 🔿 |
| Problems remembering appointments or obligations. | 🔿 | 🔿 | 🔿 | 🔿 | 🔿 |
| Avoid or delay getting started when you have a task that requires a lot of thought. | 🔿 | 🔿 | 🔿 | 🔿 | 🔿 |
| Fidget or squirm with your hands or feet when you have to sit down for a long time. | 🔿 | 🔿 | 🔿 | 🔿 | 🔿 |
| Feel overly active and compelled to do things, like you were driven by a motor. | 🔿 | 🔿 | 🔿 | 🔿 | 🔿 |

1. **Wellbeing**

How would you rate the following statements concerning your life overall?

|  | Strongly disagree | Disagree | Slightly disagree | Neutral | Slightly agree | Agree | Strongly agree |
| --- | --- | --- | --- | --- | --- | --- | --- |
| In most ways my life is close to my ideal. | 🔿 | 🔿 | 🔿 | 🔿 | 🔿 | 🔿 | 🔿 |
| The conditions of my life are excellent. | 🔿 | 🔿 | 🔿 | 🔿 | 🔿 | 🔿 | 🔿 |
| I am satisfied with my life. | 🔿 | 🔿 | 🔿 | 🔿 | 🔿 | 🔿 | 🔿 |
| So far I have gotten the important things I want in life. | 🔿 | 🔿 | 🔿 | 🔿 | 🔿 | 🔿 | 🔿 |
| If I could live my life over, I would change almost nothing. | 🔿 | 🔿 | 🔿 | 🔿 | 🔿 | 🔿 | 🔿 |

1. **Self-Care**

This past week, how many days did you do the following?

|  | Strongly disagree | Disagree | Neutral | Agree | Strongly agree |
| --- | --- | --- | --- | --- | --- |
| Got sufficient sleep | 🔿 | 🔿 | 🔿 | 🔿 | 🔿 |
| Exercised for at least 30 minutes. | 🔿 | 🔿 | 🔿 | 🔿 | 🔿 |
| Practiced muscle relaxation, yoga or meditation. | 🔿 | 🔿 | 🔿 | 🔿 | 🔿 |
| Ate foods that I know are detrimental to my health. | 🔿 | 🔿 | 🔿 | 🔿 | 🔿 |
| Got support from a network of caring people. | 🔿 | 🔿 | 🔿 | 🔿 | 🔿 |
| Expressed my emotions and discussed my problems and concerns with people close to me. | 🔿 | 🔿 | 🔿 | 🔿 | 🔿 |
| Tried to find comfort in my religion or spiritual beliefs. | 🔿 | 🔿 | 🔿 | 🔿 | 🔿 |
| Drank 2 or more alcoholic drinks. | 🔿 | 🔿 | 🔿 | 🔿 | 🔿 |
| Smoked. | 🔿 | 🔿 | 🔿 | 🔿 | 🔿 |
| Used other recreational drugs. | 🔿 | 🔿 | 🔿 | 🔿 | 🔿 |
| Stood up for myself, saying "no" when I needed to. | 🔿 | 🔿 | 🔿 | 🔿 | 🔿 |
